# Acute amivantamab-induced myocardial injury in a patient with epidermal growth factor receptor-mutant lung cancer: a first case report

**DOI:** 10.3389/fonc.2025.1708575

**Published:** 2025-12-09

**Authors:** Seiya Kaneko, Yosuke Dotsu, Tomoaki Inoue, Tetsufumi Motokawa, Tsuyoshi Yoshimuta, Mayako Mori, Toru Morikawa, Noritaka Honda, Kazumasa Akagi, Hiromi Tomono, Midori Matsuo, Hirokazu Taniguchi, Shinnosuke Takemoto, Hiroshi Mukae

**Affiliations:** 1 Department of Respiratory Medicine, Nagasaki University Graduate School of Biomedical Sciences, Nagasaki, Japan; 2 Department of Respiratory Medicine, Inoue Hospital, Nagasaki, Japan; 3 Department of Cardiovascular Medicine, Nagasaki University Graduate School of Biomedical Sciences, Nagasaki, Japan; 4 Clinical Oncology Center, Nagasaki University Hospital, Nagasaki, Japan; 5 Clinical Research Center, Nagasaki University Hospital, Nagasaki, Japan

**Keywords:** epidermal growth factor receptor, mesenchymal-epithelial transition, lung cancer, amivantamab, myocardial injury, case report

## Abstract

**Background:**

Amivantamab, a bispecific antibody against epidermal growth factor receptor and mesenchymal-epithelial transition receptors, has been approved for certain types of non-small cell lung cancer; however, it is known to cause severe adverse events. The management of such adverse events is necessary for maintaining the therapeutic efficacy of amivantamab. The frequency of cardiotoxicity caused by amivantamab is low, despite its association with a high incidence of severe adverse events. Most of the adverse events are cardiovascular events caused by thrombosis. No reports of amivantamab-induced myocardial injury have been published.

**Case presentation:**

We present the first case of drug-induced myocardial injury, detected with tachycardia but not associated with any cardiovascular events, immediately after initiating amivantamab. Echocardiography revealed a decrease in left ventricular ejection fraction and global longitudinal strain, while contrast-enhanced cardiac magnetic resonance imaging showed shortened T1 values, leading to a diagnosis of amivantamab-induced myocardial injury. Furthermore, with early detection and therapeutic interventions, we were able to continue treatment with amivantamab without interruption.

**Conclusions:**

When treating patients with amivantamab, oncologists should screen for cardiac disease-related symptoms, even in the absence of elevated cardiac serum biomarkers. Furthermore, when amivantamab-induced myocardial injury is suspected, a cardiologist should be consulted promptly, as the dysfunction may be reversible.

## Introduction

Amivantamab is a bispecific antibody that blocks the activities of epidermal growth factor receptor (EGFR) and mesenchymal-epithelial transition (MET). Amivantamab is used in combination with chemotherapy for patients with untreated non-small cell lung cancer (NSCLC) harboring an EGFR exon 20 insertion, or for those previously treated for NSCLC with a canonical EGFR mutation who experienced EGFR-tyrosine kinase inhibitor (TKI) failure (1, 2). Amivantamab-containing regimens have proven highly efficacious; however, clinical trials have reported a high frequency (32%–52%) of adverse events associated with their use (1–3). The incidence of cardiac-related toxicity caused by amivantamab has been reported to be low. Furthermore, no cases of myocardial injury in the absence of thrombotic events have been reported to date. We present the case of a 56-year-old male patient with advanced NSCLC harboring a canonical EGFR mutation who was treated with chemotherapy in conjunction with amivantamab and developed amivantamab-induced myocardial injury in the absence of thrombotic events.

## Case presentation

A 56-year-old Japanese male with no history of smoking was referred to the hospital with symptoms of exertional dyspnea. A chest X-ray revealed a right-sided pleural effusion. Aspiration revealed malignant cytology consistent with adenocarcinoma. An irregular nodule was observed in the right upper lobe of the lung on follow-up chest computed tomography (CT). Radiographic and pathologic evaluations confirmed the diagnosis of lung adenocarcinoma. Furthermore, the detection of an EGFR exon 19 deletion on genetic testing led to the initiation of oral targeted therapy with osimertinib.

**Figure 1:**
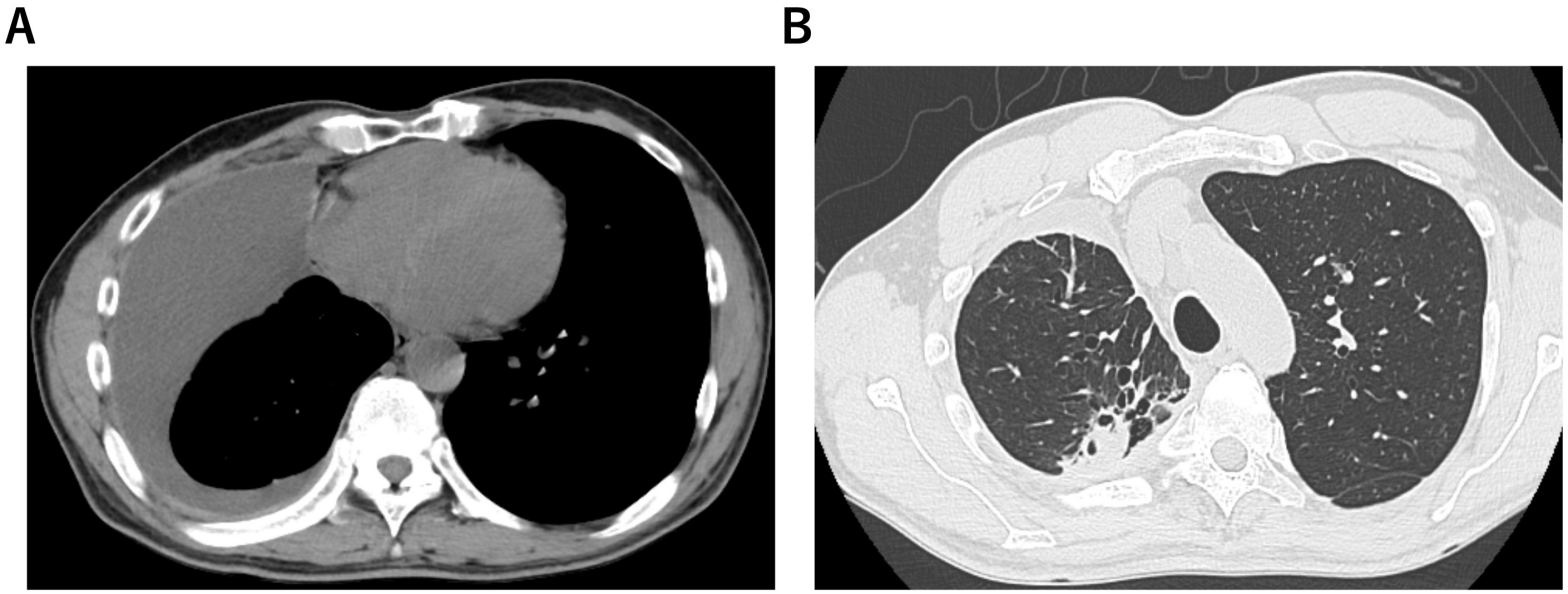
A patient with epidermal growth factor receptor-mutated non-small cell lung carcinoma. Computed tomography scans showed right pleural effusion **(A)** and nodules in the right upper lobe **(B)** before treatment with amivantamab.

**Figure 2:**
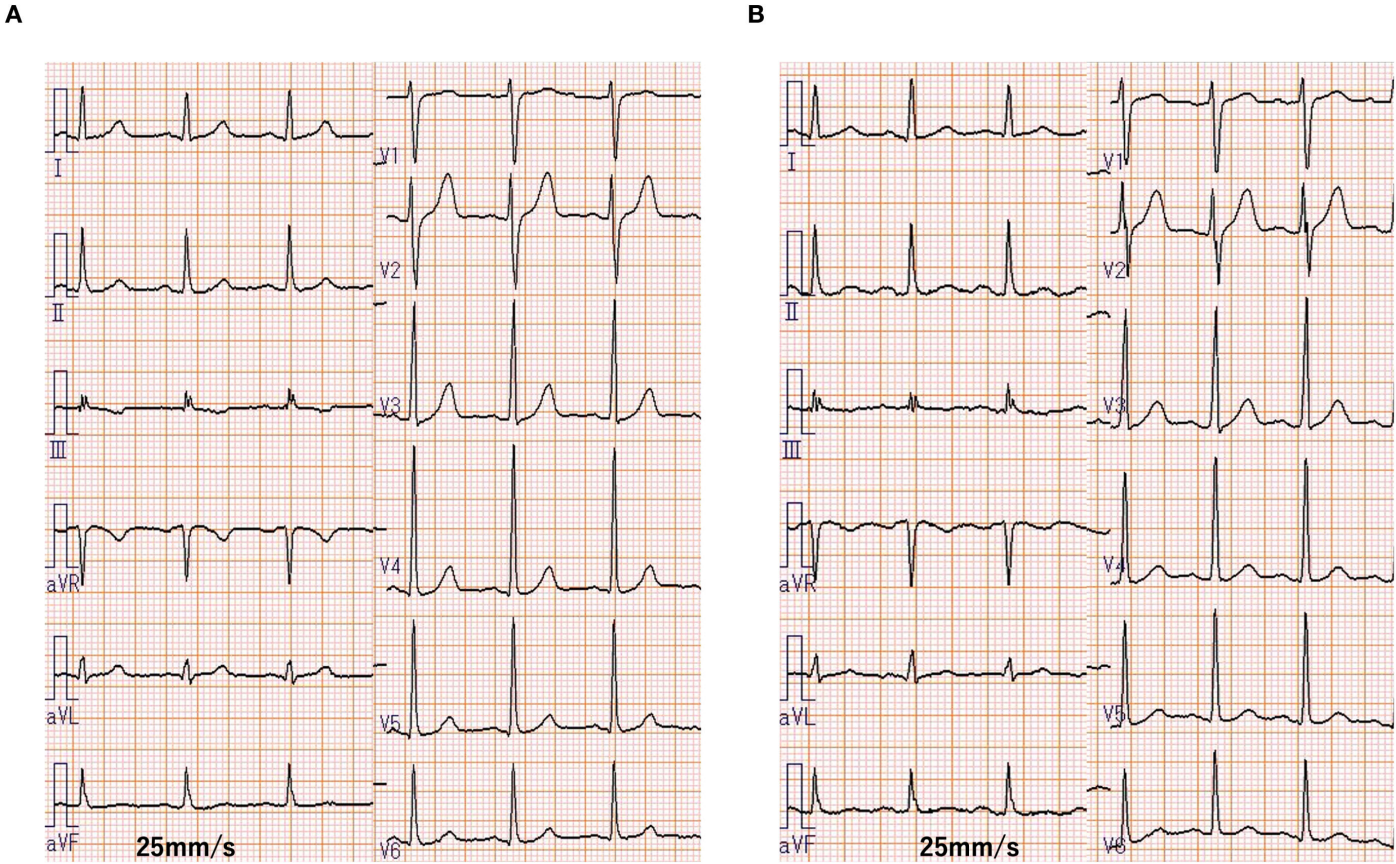
Comparison of 12-lead electrocardiograms (ECG) at baseline and after treatment. **(A)** Baseline ECG showing normal sinus rhythm (HR: 90 bpm). **(B)** ECG obtained 9 days after treatment commencement showing sinus tachycardia (HR: 108 bpm) without ischemic changes. Paper speed: 25mm/s.

After 13 months of osimertinib treatment, the level of the carcinoembryonic antigen was slightly elevated at 8.2 ng/mL (normal range <5.0 ng/mL). A chest CT scan revealed enlargement of the right upper lobe nodule and increased right pleural effusion (**Figures 1A, B**). Based on these results, second-line treatment with carboplatin, pemetrexed, and amivantamab was initiated. Electrocardiogram (ECG), serum cardiac markers, and echocardiography performed before initiation of treatment all yielded normal results (**Table 1**, **Figure 2**).

**Figure 3:**
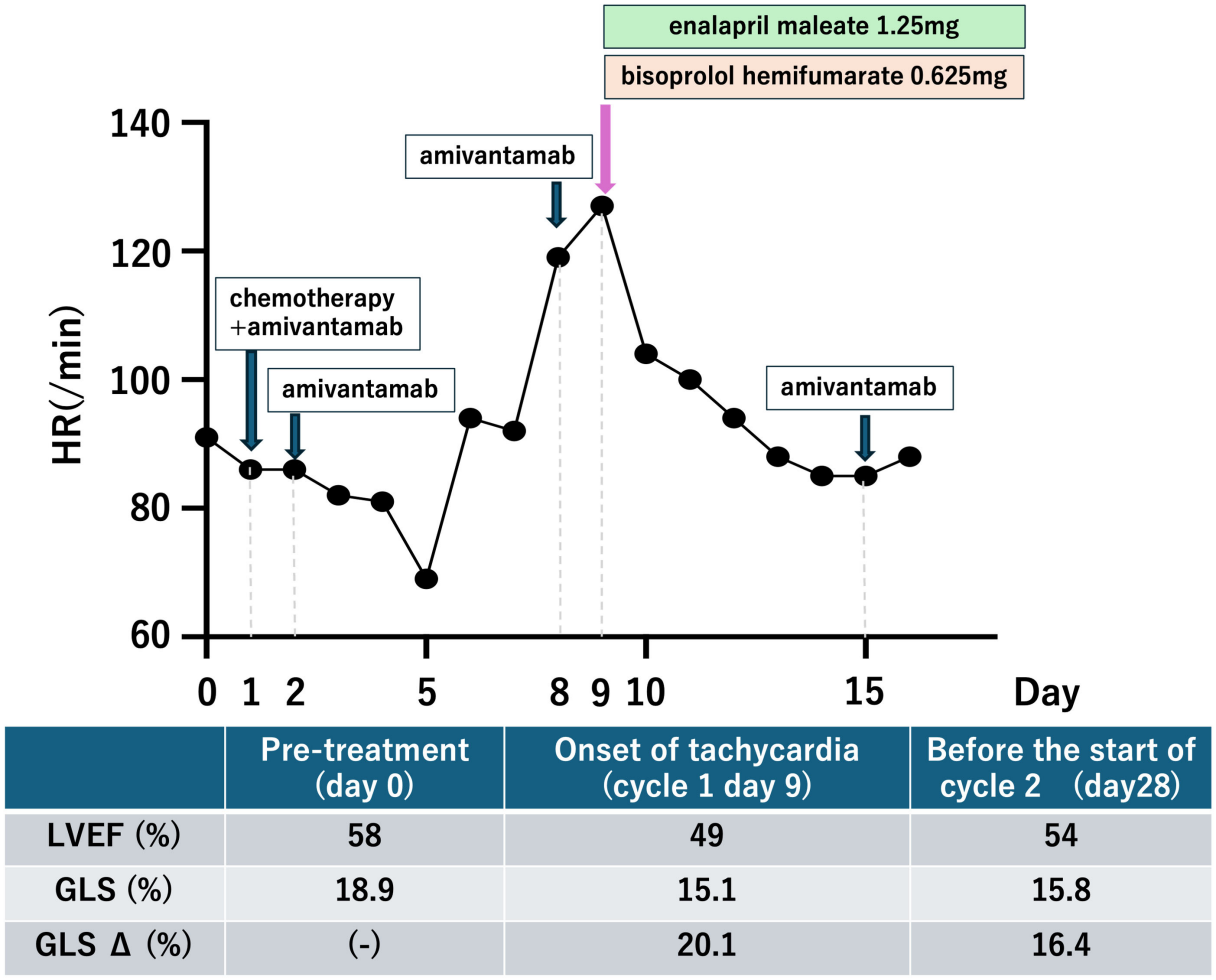
Time course of this case.

**Figure 4:**
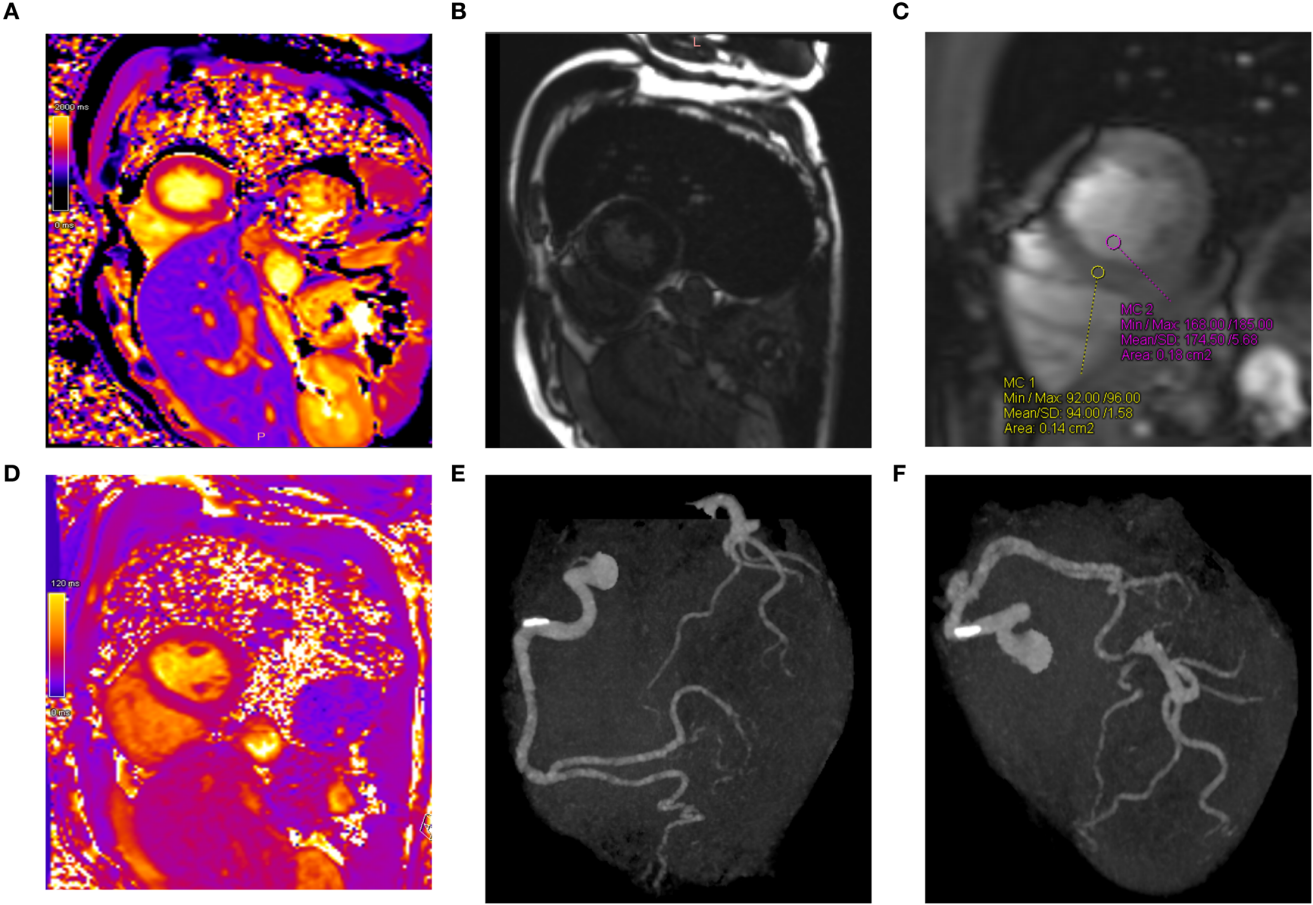
Detection of myocardial injury using cardiac imaging. **(A)** T1 mapping showed no clear elevation of native T1 values. **(B)** Delayed enhancement imaging revealed no areas of hyperenhancement. **(C)** Contrast-enhanced cardiac magnetic resonance imaging revealed areas of T1 shortening in the myocardium, indicative of drug-induced myocardial injury. **(D)** T2 mapping showed no evidence of myocardial edema. **(E, F)** No significant stenosis of the coronary arteries was observed on contrast-enhanced cardiac computed tomography.

The patient developed tachycardia 9 days following initiation of treatment, which occurred 1 day after the administration of amivantamab. ECG showed no ischemic changes, with normal sinus rhythm and sinus tachycardia at 108 bpm. Cardiac and coagulation markers, including myocardium markers, showed no elevation (**Table 1**). However, echocardiography revealed a mild reduction in left ventricular ejection fraction (LVEF) and a significant reduction in global longitudinal strain (GLS) (**Figure 3**). Furthermore, contrast-enhanced cardiac magnetic resonance imaging (MRI) revealed no apparent left ventricular wall thickening or wall motion abnormalities. Although there was no clear elevation of native T1 values or late gadolinium enhancement (**Figures 4A, B**), a shorted T1 signal on contrast imaging was observed, indicative of drug-induced myocardial injury (**Figure 4C**). Additionally, no evidence of myocardial edema was detected on T2 mapping or T2-weighted imaging (**Figure 4D**). Finally, contrast-enhanced cardiac CT showed no evidence of coronary artery disease (**Figures 4E, F**).

**Table 1:** The changes in serum cardiac markers over time with treatment.

	reference range	pre-treatment	onset of tachycardia (cycle 1 day 9)	before the start of cycle 2
CK (IU/L)	62–287	124	156	211
Troponin T (ng/mL)	≤0.014	(-)	0.005	0.007
NT-proBNP (pg/mL)	≤125	(-)	25.4	22.4
D-dimer (μg/mL)	<1.0	1.1	1.2	0.7

Following immediate consultation with cardiologists, the patient was treated with enalapril maleate 1.25 mg and bisoprolol hemifumarate 0.625 mg. The patient’s condition improved, and a follow-up echocardiogram was conducted 28 days after treatment initiation (i.e., 19 days after the event onset). LVEF had nearly returned to its baseline value, but GLS only showed a slight improvement, remaining slightly lower than the baseline value. The patient is currently receiving treatment with amivantamab under close cardiology follow-up.

## Discussion

In cancer therapy, cancer treatment-related cardiac dysfunction (CTRCD) is a potentially life-threatening adverse event. CTRCD refers to new-onset cardiac dysfunction or worsening of pre-existing dysfunction due to cancer treatment, assessed by measuring LVEF and GLS on echocardiography (4). In this case, the rapid decline in LVEF and GLS after amivantamab administration, along with exclusion of other potential causes, confirmed amivantamab-induced CTRCD. One of the main limitations of this case study lies in the fast that carboplatin and pemetrexed might have contributed to the development of myocardial injury. However, these agents rarely lead to such an outcome. For example, Quan et al. reported a case in which myocardial injury occurred in the ultra-acute phase following combination therapy with carboplatin and pemetrexed (5). In our case, carboplatin and pemetrexed were administered only on day 1, whereas amivantamab was the sole agent administered on the day prior to the onset of myocardial injury. Considering this temporal relationship, amivantamab suggestively may have played a causative role in inducing the myocardial injury. GLS, troponin T, and N-terminal pro-B-type natriuretic peptide (NT-proBNP) are considered useful indicators for the early detection of CTRCD (6–8); Although only GLS decreased in the present case, the sensitivity of troponin for detecting CTRCD has been reported to be 0.78 (9), and CTRCD induced by molecular targeted agents has been linked to functional or metabolic alterations rather than direct myocardial necrosis (10). Furthermore, the absense of correlation between NT-proBNP elevation and left ventricular dysfunction has also been reported (11). These findings suggest that negative results for troponin and NT-proBNP do not necessarily exclude the presence of CTRCD, highlighting the importance of comprehensive assessment that incorporated imaging-related findings. And post-contrast T1 shortening on cardiac MRI constituted the main imaging finding. Post-contrast T1 shortening and increased extracellular volume fraction on cardiac MRI have been reported to indicate interstitial expansion even in patients without left ventricular hypertrophy (12). These findings suggest that myocardial injury and remodeling may occur prior to overt hypertrophy or late gadolinium enhancement, and this has been reported as an imaging feature that supports the diagnosis of drug-induced myocardial injury (12). Regarding treatment, angiotensin-converting enzyme inhibitors and β-blockers have been reported to be effective for CTRCD, and early intervention may allow continuation of cancer therapy (6, 13, 14).

Several studies have reported that trastuzumab, an anti-human epidermal growth factor receptor 2 (HER2) agent, is a representative molecularly targeted therapy that can cause CTRCD (15–17). Reports have also indicated that early intervention for treating CTRCD caused by trastuzumab enables continuation of treatment (18, 19). HER2 and EGFR belong to the same erythroblastosis group B receptor family and have closely related functions (20). Furthermore, their kinase domains share approximately 83% sequence identity (21). Compared with first- and second-generation EGFR-TKIs, osimertinib is more likely to cause a decrease in LVEF (22). Although inhibition of HER2 by osimertinib has been suggested as a possible mechanism, its inhibitory activity is reported to be 6–12 times lower than that of other HER2 inhibitors such as lapatinib or afatinib, and therefore, the involvement of HER2 in this mechanism remains unclear (23). Report suggests that one possible contributing factor is the exacerbation of endoplasmic reticulum stress induced by osimertinib, which is closely associated with the onset and progression of heart failure (24). In addition to kinase inhibition, amivantamab reportedly exerts antitumor effects through Fc-mediated immune mechanisms, including antibody-dependent cellular cytotoxicity, antibody-dependent cellular phagocytosis and complement-dependent cytotoxicity (25–27). These effects involve the recruitment and activation of natural killer cells and macrophages, leading to direct tumor cell killing and modulation of the tumor microenvironment (28). Although such Fc-mediated effects have not been established for small molecule EGFR-TKIs, these findings show that the biological impact of EGFR/HER2 inhibition extends beyond kinase suppression and may involve complex immunogenic crosstalk. Given that immune activation and cytokine release can influence cardiomyocyte stress pays, Fc-dependent immune mechanisms may represent an additional factor linking EGFR/HER2-targeted therapy to CTRCD development. Taken together, these findings indicate that agents inhibiting EGFR or HER2 require careful monitoring for the development of CTRCD as highlighted in a review of cardio-oncology surveillance for targeted therapies, including the 2022 ESC Cardio-Oncology Guidelines (29).

## Conclusion

To the best of our knowledge, this is the first reported case of amivantamab-induced myocardial injury without thrombotic events. Furthermore, it is critically important to promptly consult with cardiologists if CTRCD is suspected, even in the absence of elevated cardiac serum biomarkers, to allow continuation of amivantamab-containing treatments for better outcomes, as this CTRCD may be reversible.

## Data Availability

The original contributions presented in the study are included in the article/**Supplementary Material**. Further inquiries can be directed to the corresponding author.

## Supplementary material

Supplementary Video 1 Baseline echocardiographic findings showing normal left ventricular contraction.

Supplementary Video 2 Echocardiographic findings at the onset of myocardial injury demonstrating reduced left ventricular ejection fraction and wall-motion abnormalities.

Supplementary Video 3 Speckle-tracking echocardiographic analysis from standard apical **(A)** 4-, **(B)** 2-, **(C)** 3-chamber views showing the shortening of global longitudinal strain at the onset of myocardial injury.
